# Development of a novel diagnostic assay for insulin receptor autoantibodies based on a patient with autoimmune hypoglycaemia

**DOI:** 10.3389/fendo.2022.1029297

**Published:** 2022-10-25

**Authors:** Leiluo Geng, Cheuk-Lik Wong, Boya Liao, Ying Lin, Hao Han, Karen S. L. Lam, Aimin Xu, Chi-Ho Lee, Vicki H. K. Tam

**Affiliations:** ^1^ State Key Laboratory of Pharmaceutical Biotechnology, The University of Hong Kong, Hong Kong, Hong Kong SAR, China; ^2^ Department of Medicine, School of Clinical Medicine, The University of Hong Kong, Hong Kong, Hong Kong SAR, China; ^3^ Diabetes Centre, Department of Medicine and Geriatrics, Caritas Medical Centre, Hong Kong, Hong Kong SAR, China; ^4^ School of Chinese Medicine, Hong Kong Baptist University, Hong Kong, Hong Kong SAR, China; ^5^ Department of Pharmacology and Pharmacy, The University of Hong Kong, Hong Kong, Hong Kong SAR, China

**Keywords:** autoimmune hypoglycaemia, insulin receptor autoantibodies, type B insulin resistance, diagnosis, ELISA

## Abstract

Differential diagnosis of hypoglycaemia can at times be challenging for patients who appear to be well. Here we identify the case of a 66-year-old Chinese man presenting with recurrent episodes of fasting hypoglycaemia and confusion without any other manifestations. He had no personal or family history of diabetes, nor was he on any hypoglycaemic drugs. The fasting insulin levels were elevated while the C-peptide and pro-insulin levels were slightly low or normal. Antibodies against insulin were negative and levels of insulin-like growth factors were normal. A series of imaging diagnosis excluded the presence of insulinoma or ectopic insulin-secreting neuroendocrine tumor. Ultimately, insulin receptor autoantibodies (IRAb) were detected by both immunoprecipitation assay and enzyme-linked immunosorbent assay (ELISA) developed in house. In a cell study, the immunoglobulins isolated from this patient exerted insulin-like effects on stimulation of post-insulin receptor signaling and glucose uptake as well as inhibited ^125^I-insulin binding with insulin receptors. Collectively, this patient was diagnosed with IRAb-induced autoimmune hypoglycaemia. Although this patient had no obvious immune disorders, several autoantibodies were identified in his plasma samples, suggesting the patient might have mild aberrant autoimmunity and therefore generated IRAb. IRAb-related disease is uncommon and possibly underdiagnosed or missed due to the lack of simple detection methods for IRAb. Our in-house user-friendly ELISA kit provides a valuable tool for diagnosis of this disease.

## Introduction

Patients with insulin receptor autoantibodies (IRAb) were firstly recognized by Kahn et al. in 1976 (1). Since then, about 119 patients with IRAb have been identified and reported throughout the world (2). IRAb represents a heterogenous group of polyclonal antibodies, which can bind with the insulin receptors on cell surface to disrupt or block the normal functions of insulin (3). While patients with IRAb classically have type B insulin resistance syndrome (TBIRS) presenting with severe insulin resistance and refractory hyperglycaemia (4), there have also been reports of patients with spontaneous hypoglycaemia as their only clinical manifestation (5).

However, due to the lack of a simple, reliable and commercially available IRAb detection assay, the diagnosis of IRAb-mediated hyper-or hypoglycaemia can sometimes be difficult. Here, we identified a Chinese man who presented with recurrent episodes of spontaneous hypoglycaemia. After extensive workup, this patient was eventually diagnosed to have rare autoimmune hypoglycaemia caused by high titers of IRAb. To facilitate the diagnosis of IRAb-related diseases, we developed an enzyme-linked immunosorbent assay (ELISA) for IRAb measurement that was user-friendly, with a sensitivity comparable to conventional immunoprecipitation (IP) assay and a specificity validated in healthy individuals showing negative results. To our knowledge, this ELISA kit is also the first diagnostic tool for IRAb developed with *in-vitro* functional validation, and can be used in most clinical laboratories without the requirement of any special reagents or apparatus.

## Clinical case

A 66-year-old Chinese man was admitted for recurrent episodes of confusion and an increase in mental dullness in August 2020, especially during fasting in the early morning and before lunch. One morning, while walking in the shopping center, his wife noticed that the patient had mental unawareness with twitching of one of his lower limbs that lasted for several minutes. He has no known drug allergy and was on warfarin, allopurinol, colchicine, digoxin, enalapril, frusemide and potassium chloride prior to his admission. He denied taking any over-the-counter medications or herbal medicine. He reported good appetite and had weight gain of 3-4 kg over the past few months. The patient had a history of Graves’ hyperthyroidism diagnosed in 2014. His anti-thyroglobulin antibody (Anti-Tg) and anti-thyroid peroxidase antibody (Anti-TPO) levels were 6750 units (Reference < 101) and 922 units (Reference < 101), respectively. He received a course of carbimazole from May 2014 to December 2015, and remained in remission since then without the need of definitive therapy. On admission, his thyroid-stimulating hormone (TSH) and free T4 levels were both normal. Except for the history of Graves’ disease, his past health was unremarkable and he did not have history of diabetes. On physical examination, his body mass index (BMI) was 25.3 kg/m^2^. There was no skin hyperpigmentation or acanthosis nigricans. His plasma glucose on admission was 3.0 mmol/L and urine toxicology screening was negative for sulphonylureas. His hemoglobin A1c (HbA1c) was 5.1%. A prolonged fasting test was therefore performed for spontaneous hypoglycaemia. After only 6 hours since the test began, the patient experienced symptomatic hypoglycaemia with plasma glucose level went down to 1.6 mmol/L, and his concomitant serum insulin level was 53 mIU/L and C-peptide level was 0.23 nmol/L and pro-insulin level was 3.8 pmol/L. The insulin to C-peptide molar ratio was elevated at 1.6 (N: 0.03-0.25). Factitious hypoglycaemia was excluded since the patient was unlikely to access insulin products under close supervision in our hospital. His serum beta-hydroxybutyrate level was <0.1 mmol/L during hypoglycaemia. Glucagon stimulation test revealed a rise of plasma glucose from 2.2 mmol/L to 3.7 mmol/L. Subsequent investigations including serum insulin-like growth factors (IGF-1 and IGF-2) were normal (**Table 1**). The overall picture was suggestive of endogenous hyperinsulinaemic hypoglycaemia. However, radiological investigations including computerized tomography of the pancreas, endoscopic ultrasound, ^68^Gallium-DOTATATE- and ^18^FDG-PET-CT scans did not reveal any evidence of insulinoma except for multiple hypermetabolic lymph nodes over his bilateral jugular, bilateral axillary, left supraclavicular fossa, mediastinal, para-aortic and bilateral iliac regions commented to be reactive lymphadenopathy. He was then tested for antibodies against insulin but the results were also negative. Eventually, we measured his circulating levels of IRAb, and confirmed the diagnosis of autoimmune hypoglycaemia caused by IRAb.

**Table 1:** Glycaemic control parameters and autoantibodies of the patient on admission.

Parameter	Result	Reference
Fasting blood glucose (mM)	1.6	4.0-5.9
HbA1c (%)	5.1	4.0-5.6
Fasting insulin (mIU/L)	53	2.6-24.9
Fasting C-peptide (nM)	0.23	0.26-0.62
Fasting insulin/C-peptide molar ratio	1.6	0.03-0.25
Fasting pro-insulin (pM)	3.8	3.6-22
Fasting BOHB (mM)	<0.1	<0.3
IGF-1 (μg/L)	34	41-279
IGF-2 (μg/L)	125	100-200
Anti-insulin antibody	Negative	Negative
GAD65 autoantibody (IU/mL)	52.3	<5.0
Anti-nuclear antibody	1:320	>1:80
Anti-dsDNA antibody	Negative	Negative
Anti-extractable nuclear antigen antibody	Negative	Negative
Coombs’ test	Positive	Negative
Rheumatoid factor	Negative	Negative
Anti-neutrophil cytoplasmic antibody	Negative	Negative
Anti-cardiolipin antibody	Negative	Negative
Lupus anticoagulant	Negative	Negative

HbA1c, hemoglobin A1c; BOHB, β-hydroxybutyrate; IGF-1/2, insulin-like growth factor 1/2; GAD65, glutamic acid decarboxylase 65-kilodalton isoform; dsDNA, double-stranded DNA.

The presence of IRAb has been reported to be associated with multiple myeloma, lymphoproliferative and autoimmune diseases such as systemic lupus erythematosus (6–9). In this patient, mild thrombocytopenia with platelet count of around 70-100 ×10^9^/L was noted. However, his serum protein electrophoresis did not reveal any paraproteinaemia, and his bone marrow examination showed normocellular marrow with mild megakaryocytic hyperplasia suggestive of peripheral consumption. Although the patient did not have family history of autoimmune diseases, he was positive for anti-nuclear antibody (ANA) at a titre of 1:320 and direct Coombs’ test. However, his rheumatoid factor, anti-dsDNA, anti-extractable nuclear antigen, anti-cardiolipin and anti-neutrophil cytoplasmic antibodies were all negative. His C3 and C4 levels were 0.44 g/L (N: 0.9-1.8 g/L) and <0.04 (N: 0.1-0.4 g/L) respectively. Interestingly, his autoantibody against glutamic acid decarboxylase 65-kilodalton isoform (GAD65) was also positive at a titre of 52.3 U/mL (N: <5.0 U/mL). His thyroid function and low dose short Synacthen® test were otherwise normal (**Table 1**).

**Figure 1:**
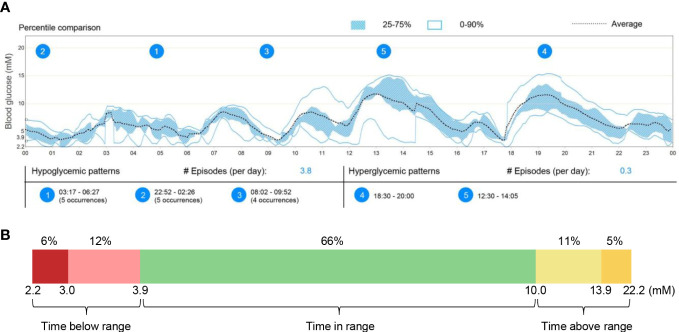
Continuous glucose monitoring in this patient from September 25-30, 2020. **(A)** Averaged and percentile blood glucose levels during this 6-day continuous monitoring. The patterns and occurrences of hypoglycaemic and hyperglycaemic episodes were provided. **(B)** Quantification of time below range (2.2-3.9mM), time in range (3.9-10.0mM), and time above range (10.0-22.2mM) during the whole glucose monitoring.

The patient underwent a 6-day continuous glucose monitoring and revealed significant post-prandial hyperglycaemic excursions in addition to multiple hypoglycaemic episodes occurring mostly during the overnight periods (22:00-6:00) (**Figure 1A**). The average time in range was only 66% while the time below range was as high as 18% (**Figure 1B**).

After the diagnosis of autoimmune hypoglycemia due to IRAb, we started the patient on high dose prednisolone (30 mg daily). Within 3-4 weeks after starting prednisolone, there was significant improvement in midnight hypoglycaemia and reduction of his IRAb levels. However, in view of fluid retention, the dosage of prednisolone was reduced to 15 mg daily after 1 month. Since then, the patient had recurrent hypoglycaemic symptoms with accompanying increase in IRAb levels during this period. The patient subsequently received a few courses of intravenous immunoglobulin (IVIg), which showed only a transient response. Another month later, the patient was admitted for severe hypoglycaemia resulting in a fall with head injury. He was then added mycophenolate mofetil (MMF) 500 mg twice daily and continued with a lower prednisolone dose of 7.5 mg daily. The patient responded well with less hypoglycaemic symptoms, reduction in IRAb level, as well as the requirement of taking raw corn starch at midnight. However, he was admitted eight months later for septic shock due to methicillin-sensitive Staphylococcus aureus (MSSA) septicaemia. He was diagnosed with infective endocarditis and spondylodiscitis. He required inotropic support in intensive care unit and prolonged antibiotic treatment. MMF was then stopped due to infective risk. The patient developed severe midnight hypoglycaemia again, which was only improved by a maintenance dose of prednisolone of at least 7.5 mg daily. At 9 months after his initial presentation, the patient was eventually free of further neuroglycopenic symptoms and his HbA1c level rose to 6.7%.

## Materials and methods

### Immunoprecipitation assay for IRAb detection

**Figure 2:**
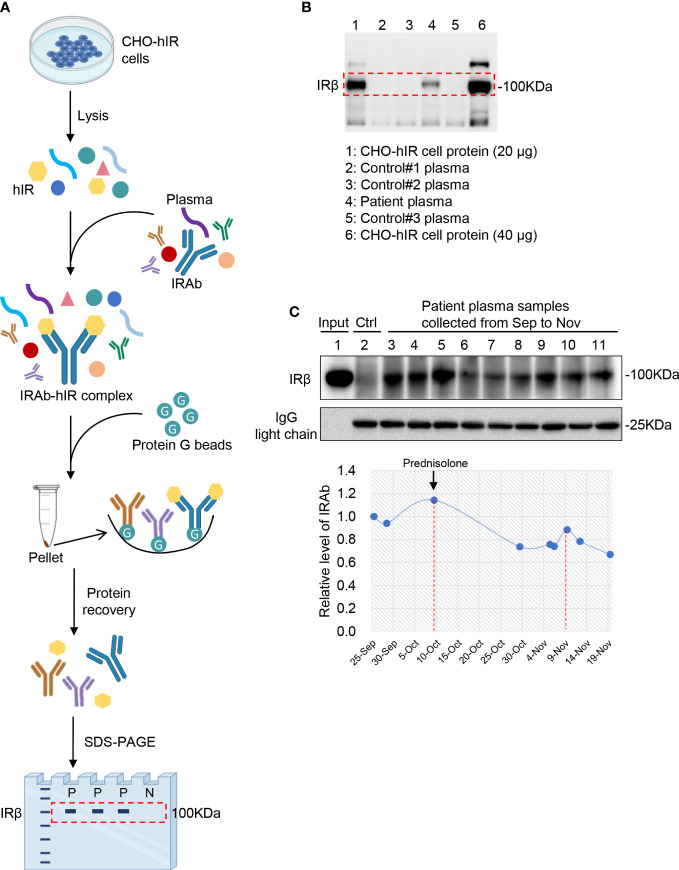
Detection of IRAb in the patient plasma. **(A)** Diagram showing the principle and procedures for immunoprecipitation assay for IRAb detection. **(B)** Human insulin receptor protein was detected in the precipitates from patient plasma sample (lane 4) but not from three control plasma samples (lane 2, 3, 5). CHO-hIR cell lysates were loaded as positive control (20µg total protein for lane 1 and 40µg total protein for lane 6). Similar results were obtained from another two independent experiments. IRβ, insulin receptor β subunit. **(C)** IRAb titers were dynamically monitored in this patient from September to November. The relative IRAb titers were calculated by densitometric quantification of IRβ levels and normalized to levels of plasma IgG light chain. Representative result from three independent experiments was shown.

The principle of IRAb detection by IP assay is shown in **Figure 2A**. First, total protein was extracted from CHO-hIR (ATCC, #CRL-3307™) cells with stable expression of human insulin receptors using cell lysis buffer (150 mM NaCl, 1.0% NP-40, 0.5% sodium deoxycholate, 0.1% SDS, 50 mM Tris-HCl, pH 8.0) containing protease inhibitors cocktail (Roche, #04693159001), followed by protein concentration quantification by BCA kit (Pierce, #23227). Secondly, 2 μL plasma was mixed with 60 μg CHO-hIR cell lysate protein in 300 μL RIPA250 buffer (50 mM Tris-HCl pH 7.4, 250 mM NaCl, 1% NP40, 0.5% sodium deoxycholate), followed with mixing at 4°C overnight on a rotating incubator (Thomas Scientific, Swedesboro). Thirdly, 25 μL protein G beads (Cytiva, #17061805) were added and incubated for 3 hours at 4°C in the next day. At last, the beads were centrifuged at 2000 rpm for 5 minutes (min) and washed with 600 μL RIPA250 buffer for 5 times. Protein loading buffer (4% SDS, 10% 2-mercaptoethanol, 20% glycerol, 0.004% bromophenol blue, 125 mM Tris-HCl, pH 6.8) was added and boiled at 99°C for 8 min to release the protein from the protein G beads. The released protein was subjected to SDS-PAGE and insulin receptors were detected with commercial rabbit anti-insulin receptor β antibody (CST, #3025) by immunoblotting.

### Purification of immunoglobulins from plasma

Approval by institutional review boards was obtained for the studies. Written informed consent was obtained from the patient before blood collection for IRAb measurements. On the other hand, plasma samples from healthy individuals were retrieved from our established bio-bank. 1 mL plasma was mixed with 0.5 mL protein G beads and 8.5 ml phosphate-buffered saline (PBS, pH 7.4) and allowed to equilibrate overnight at 4°C. In the next day, the mixture was carefully poured into a column, followed by washing with 50 mL PBS. The bound immunoglobulins were eluted with 1 mL of 0.2 mol/L glycine-HCI buffer (pH 2.8), which was repeated for another five times. At last, the elute in the collection tube was immediately neutralized with 250 μL 1 mol/L NaOH solution. The immunoglobulins were dialyzed and then concentrated in PBS. The protein concentration was determined by a BCA kit (Pierce, #23227) and stored at -80°C for the following experiments.

### Cell study

CHO-hIR (ATCC, #CRL-3307™) cells were maintained in Ham’s F12 medium (Gibco, #21700075) supplemented with 10% fetal bovine serum (Gibco, #A3160801) and 0.38 mg/mL hygromycin B (Invitrogen, # 10687010) at 37°C with 5% CO_2_. The cells were fasted with plain medium for 4 hours before treatment with immunoglobulins extracted from plasma samples of the patient or the healthy control. For the time-course study, the cells were treated with 500 μg/mL immunoglobulins for 0, 5, 10, 20, and 40 min, followed by cell harvest. For the dosage gradient study, the cells were treated with 0, 50, 500, and 1000 μg/mL immunoglobulins for 15 min, followed by cell harvest. Cells treated with PBS or 100 nM insulin (Actrapid HM) were included as negative or positive controls. Total protein was extracted from the harvested cells and subjected to immunoblotting for insulin receptor downstream signals. For glucose uptake assay, confluent CHO-hIR cells were fasted for 4 hours with plain medium and treated with 500 μg/mL Patient Ig, 500 μg/mL Control Ig, PBS or 100 nM insulin for 30 min, followed by measurement of glucose uptake capacity with a commercial kit (Abcam, #ab136955).

### Immunoblotting analysis

Total protein was isolated from cells using RIPA buffer (150 mM NaCl, 1.0% NP-40, 0.5% sodium deoxycholate, 0.1% SDS, 50 mM Tris-HCl, pH 8.0) containing protease inhibitors cocktail (Roche, #04693159001), resolved by SDS-PAGE, and then transferred to PVDF membranes (Millipore, #IPVH00010). The membranes were then blocked with 10% milk for 2 hours at room temperature and incubated with primary antibodies (1:2000, p-Akt Ser473, CST #9271; t-Akt, CST #9272; p-Erk1/2 Thr202/Tyr204, CST #9109; t-Erk, CST#9102; HSP90, CST #4874; human IgG light chain, Abcam #ab124727) overnight at 4°C. In the next day, after washing with PBST buffer for 5 times, the membranes were incubated with anti-rabbit HRP-conjugated secondary antibodies (1:3000, CST #7074) for 45 min at room temperature, followed by intensive washing with PBST buffer for 5 times. Finally, the protein bands were visualized with SignalFire™ ECL Reagent (CST, #6883) under the ChemiDocTM MP Imaging System (Bio-Rad) and quantified with the ImageJ software.

### Insulin-binding-inhibition study

The experiment was performed according to a protocol as previously described with minor modifications (3). In brief, the confluent CHO-hIR cell monolayers in a 24-well plate (2.3 ×10^5^ cells/well) were washed with 0.8 mL PBS and incubated in duplicates with various concentrations of immunogloblulins from the patient or the healthy control in a final 0.5 mL binding buffer (100 mM HEPES, 120 mM NaCI, 1 mM MgSO_4_, 1 mM EDTA, 10 mM glucose, 1.5 mM sodium acetate, and 0.1% bovine serum albumin, pH 8.0) for 2 hours at 22°C. The binding buffer was then aspirated, and the cells were washed with 0.8 mL PBS and incubated with 0.01 μCi of ^125^I-insulin (PerkinElmer, #NEX420050UC) in a final 0.5 mL binding buffer per well for 1 hour at 15°C. The incubation was terminated by washing twice with cold PBS and adding 1 mL of 1 M NaOH. Cell-associated radioactivity was counted in 4 mL scintillation fluid (American Biosciences, #NACS104) with Packard Cobra II Auto Gamma Counter (GMI, Ramsey). The cells incubated with only binding buffer but without immunoglobulins were included as blank control and reference group.

### Statistical analysis

All statistical analysis was performed using GraphPad (GraphPad Prism 8.4.3) and Microsoft Excel. Values were expressed as mean ± standard deviation (SD) as specified in each figure. Comparisons between groups were determined by *t*-test or analysis of variance (ANOVA). Pearson’s correlation analysis was used to calculate the association between two factors. In all analysis, a two-sided p<0.05 was considered statistical significance.

## Results

### Presence of circulating IRAb with dynamic changes in the patient

In the first plasma sample at diagnosis, a clear band for the insulin receptor protein was detected after immunoprecipitation of the patient plasma with CHO-hIR cell protein, but not that from the control samples (**Figure 2B**). Similar results were obtained from another two independent experiments. This IP assay was also performed without adding the CHO-hIR cell lysate, but nothing was detected on the gel using either patient or control plasma samples (data not shown). These results confirmed the presence of circulating IRAb in this patient. Moreover, when comparing his IRAb titers serially based on the densities of insulin receptor protein bands, his IRAb titers gradually decreased after prednisolone treatment but rose again afterwards (**Figure 2C**), in keeping with the usual serum half-life of human endogenous antibodies of around 10-21 days (10).

### IRAb acts as an agonist for the insulin receptor to stimulate glucose uptake in vitro

**Figure 3:**
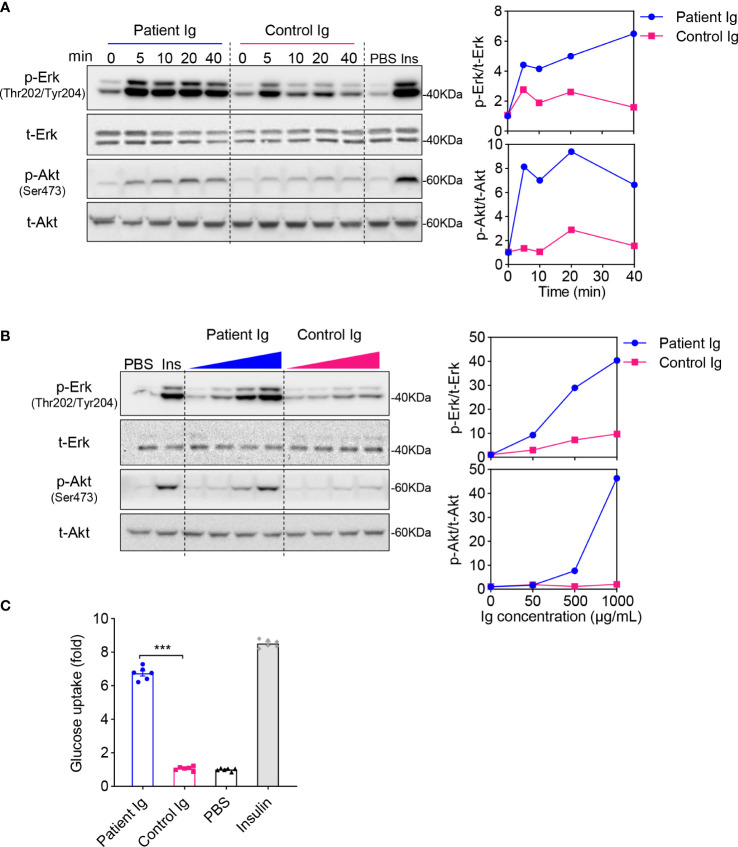
Immunoglobulins extracted from patient plasma exert insulin-like effects in CHO-hIR cells. **(A, B)** Confluent CHO-hIR cells were fasted for 4h, followed with treatment of 500 μg/mL immunoglobulins (Ig) for 0, 5, 10, 20, and 40 min **(A)** or treatment of 0, 50, 500, and 1000 μg/mL Ig for 15 min **(B)**. Treatment of PBS or 100 nM insulin was included as negative or positive control. p-Erk(Thr202/Tyr204), t-Erk, p-Akt(Ser473) and t-Akt were measured by immunoblotting. The relative values of p-Erk/t-Erk and p-Akt/t-Akt were quantified in the groups of Patient Ig and Control Ig separately. Representative results from three independent experiments were shown. **(C)** Glucose uptake was measured in CHO-hIR cells treated with 500 μg/mL Patient Ig, 500 μg/mL Control Ig, PBS or 100 nM insulin for 30 min (n=6, ***p<0.001).

Based on the clinical presentation of this patient, it is likely that his IRAb had activated the insulin receptor and its downstream signaling, which induced excessive glucose uptake leading to hypoglycaemia. To test this hypothesis, post-insulin receptor signaling in the CHO-hIR cells was examined after they were treated with immunoglobins extracted from his plasma. Consistently, we found that IRAb, which was present among the immunoglobins extracted from the patient, significantly stimulated phosphorylation of Akt and Erk more than the immunoglobins isolated from the healthy controls in which IRAb were absent. Both the time-course and dosage gradient studies demonstrated similar results (**Figures 3A, B**). Immunoglobins from the patient induced almost 6-fold increase in glucose uptake compared to those from the healthy controls (**Figure 3C**). Collectively, these findings suggested that IRAb from this patient could activate insulin receptors and induce glucose uptake similarly as insulin.

### IRAb inhibits the binding of insulin with insulin receptor

**Figure 4:**
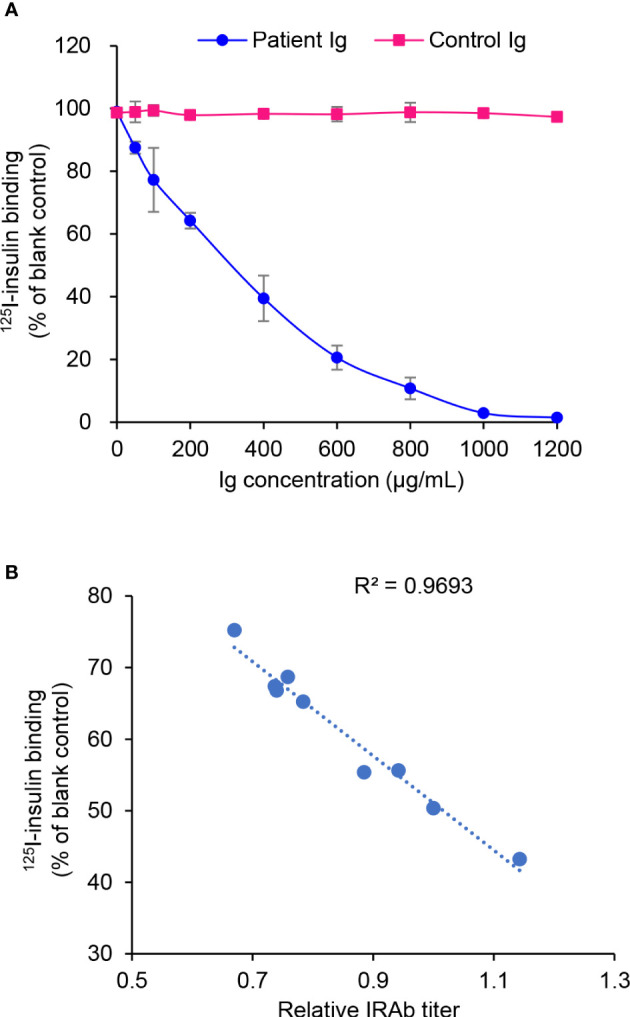
Inhibition of ^125^I-insulin binding with CHO-hIR cells by patient immunoglobulins. **(A)** CHO-hIR cells were pre-incubated with varying concentrations of Patient immunoglobulins (Ig) or Control Ig, followed by ^125^I-insulin binding assay. CHO-hIR cells without Ig pre-incubation were included as blank control and reference group. For each Ig concentration, three duplicates were set. **(B)** Ig were extracted from patient plasma samples collected on different dates and their inhibitory effects on ^125^I-insulin binding were assessed at the concentration of 400 μg/mL. The IRAb titers were measured in these plasma samples by immunoprecipitation assay. The correlation of ^125^I-insulin binding and IRAb titer was calculated (n=9).

In this patient, despite the significantly elevated serum insulin level during hypoglycaemia, his C-peptide level was not excessively high (**Table 1**). Since insulin degradation is initiated by receptor-mediated endocytotic uptake *via* its binding with the insulin receptor on cell surface (11), we hypothesized that IRAb may interfere the binding of insulin with insulin receptors and therefore cause impaired insulin degradation and hyperinsulinaemia. To test this hypothesis, the effects of IRAb-containing immunoglobins on insulin binding with insulin receptor in CHO-hIR cells were evaluated. We found that the levels of isotope-labeled insulin in the binding assay decreased with increasing concentrations of IRAb-containing immunoglobins pre-incubated with the CHO-hIR cells (**Figure 4A**), whereas immunoglobins from the healthy controls had no obvious effects on insulin binding with insulin receptors in cells (**Figure 4A**). Moreover, the inhibitory effects on insulin-binding also positively correlated with the IRAb titers (**Figure 4B**). Taken together, these *in-vitro* findings confirmed that IRAb could inhibit insulin binding with insulin receptors in cells, suggesting an additional potential mechanism for the hyperinsulinaemia found in this patient.

### Development and verification of an easy-to-use ELISA kit for IRAb detection

**Figure 5:**
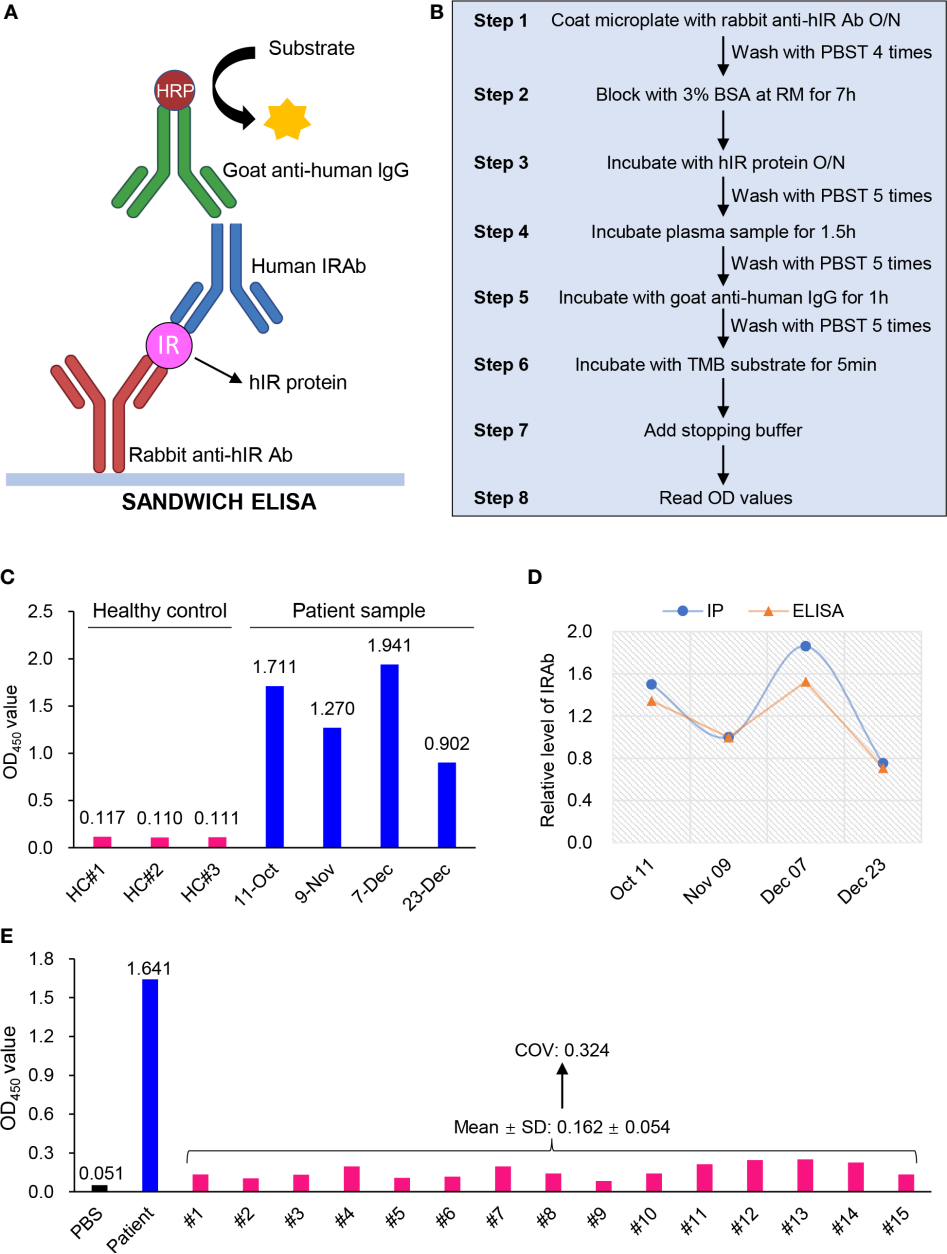
Development and validation of an ELISA kit for IRAb detection. **(A)** Diagram showing the sandwich ELISA principle for IRAb detection. **(B)** Summarized scheme for the preparation of IRAb ELISA kit and the measurement procedures. **(C)** The OD_450_ values for IRAb detection by ELISA kit in three healthy controls and this patient. The patient plasma samples were collected from Oct 11 to Dec 23, 2020. **(D)** Quantification of relative IRAb titers by immunoprecipitation (IP) assay and ELISA in the same patient plasma samples collected from Oct 11 to Dec 23, 2020. **(E)** The OD_450_ values for IRAb detection by ELISA kit in 15 healthy controls. PBS and patient sample as blank control and positive control. COV, cut-off value. SD, standard deviation.

Over the years, IP assay has been the conventional method for IRAb detection, although it is complicated, labor intensive, time-consuming and is not commonly available in most hospital laboratories due to the lack of required reagents or apparatus (12). Therefore, we developed a user-friendly ELISA kit for IRAb detection based on the sandwich-ELISA principle. The insulin receptor protein, the antigen, is bound between the capture antibody and IRAb in the sample, which is further detected by anti-human IgG secondary antibody (**Figure 5A**). As for the detailed ELISA procedures (**Figure 5B**), flat-bottom polystyrene Stripwell^TM^ microplates (Costar, #42592) were coated with 100 μL/well of 0.5 μg/mL rabbit anti-human insulin receptor antibody (made in house) in PBS (pH 7.4) overnight at 4°C, followed by washing with 200 μL/well of PBST for 4 times. The plates were then blocked with 200 μL/well of 3% BSA in PBST for 7 hours at room temperature. After that, 0.1 μg purified human insulin receptor protein (made in-house) diluted in 100 μL assay buffer (1.5% BSA in PBST) was added in each well. After incubation at 4°C overnight, almost 63±3% of insulin receptor protein was captured by the insulin receptor antibody at the bottom of the well. The plate was intensively washed with 300 μL/well of PBST for 5 times and would then be ready for use. The plate could be stored at 4°C for at least 2 weeks. On the day of the assay, 2 μL plasma sample diluted in 98 μL assay buffer was added into each well, followed by incubation at room temperature with shaking at 180 rpm for 1.5 hours. The plate was then washed with 300 μL/well PBST for 5 times, followed by incubation with goat anti-human IgG secondary antibody (1:100 in assay buffer, Invitrogen #62-8400) for 1 hour at room temperature with shaking at 180 rpm. Subsequently, the plate was washed with 300 μL/well PBST for 5 times, followed by incubation with 100 μL/well of TMB substrate (ThermoFisher, #N301) for 5 min at room temperature and terminated with 100 μL stopping buffer (1M sulfuric acid). The optical density (OD) values were read at 450 nm within 15 min.

For this ELISA kit, the averaged OD_450_ value for the four patient samples collected from October 11 to December 23 of 2020 was 1.456, while that for the three control samples was 0.112, leading to a high signal-noise ratio (S/N) of 12.98 (**Figure 5C**). After quantification, the ELISA obtained a similar result when compared with IP assay for IRAb measurements (*t*-test, p=0.173) (**Figure 5D**). Notably, we found that the changes in IRAb titers were in parallel with the overall disease course and treatment response of the patient. On the other hand, when the plasma samples from the 15 healthy donors, who were tested negative for IRAb by IP assay, were measured for IRAb using our ELISA kit, the OD_450_ reactivity varied from 0.084 to 0.251, with a mean value of 0.162 and SD 0.054. Based on these findings, a cut-off value (COV) with 3SD (mean+3SD) was determined to be 0.324 (**Figure 5E**) (13), and will be used to determine that the serum sample is negative for IRAb. We also tested the specificity of this ELISA kit using plasma samples containing insulin antibodies and all the results were negative (data not shown), suggesting the absence of cross-reactivity with anti-insulin antibodies. These preliminary testing indicated that our newly-developed ELISA kit has comparable sensitivity with IP assay and the positive samples can be easily differentiated from negative controls according to the high S/N value.

## Discussion

Autoimmune hypoglycaemia due to IRAb is an extremely rare autoimmune-metabolic disorder (5, 14–16). Although some cases have been reported, the diagnostic workup remains challenging, which is partly related to the lack of a commercially available assay for IRAb measurements. We reported a rare Chinese patient with autoimmune hypoglycaemia due to high titers of IRAb detected by conventional IP assay and our newly-developed in-house ELISA. Notably, his circulating IRAb titers changed dynamically with his clinical presentation. Moreover, with further *in-vitro* functional studies using the immunoglobulins isolated from this patient, we found that his IRAb possessed insulin-like effects and disrupted the normal function of endogenous insulin by activating post-insulin receptor signaling and stimulating glucose uptake in CHO-hIR cells, as well as inhibiting ^125^I-insulin binding with CHO-hIR cells. These *in-vitro* findings have also provided mechanistic insights into the clinical presentation of this patient. Most importantly, we have demonstrated the clinical utility of our newly developed ELISA kit for IRAb measurement, which is different from the conventional, complicated IP assay. This user-friendly ELISA can also be used in most clinical laboratories for the diagnosis of TBIRS and monitoring of treatment-responses, both of which have been clinically difficult previously.

While patients with IRAb classically have TBIRS presenting with severe insulin resistance and hyperglycaemia requiring high doses of insulin therapy (4), more than 10% of patients with IRAb present with autoimmune hypoglycaemia alone. To our knowledge, 36 cases have been reported to date since 1978 with autoimmune hypoglycaemia due to IRAb (**Supplemental Table 1**). There is no age or ethnic preponderance. Interestingly, although our patient had a personal history of Graves’ disease, IRAb has not been reported in patients with Graves’ disease, in contrast to the close association between Graves’ disease and Hirata’s disease caused by insulin autoantibodies (17, 18). The presence of IRAb has been associated with Hodgkin’s disease and autoimmune diseases, in particular systemic lupus erythematosus which had been found to be present in 46% of TBIRS patients (4, 17). Aberrant autoimmunity may induce the generation of IRAb, but how rare IRAb are produced in Hodgkin’s disease remains unknown (6, 8, 19). Accordingly, our patient also underwent comprehensive investigations for the presence of underlying autoimmune and haematological diseases, and found that the patient might have an associated lupus-like disease with positive ANA and Coombs’ test, low complement levels, immune thrombocytopenic purpura and reactive lymphadenopathy. Although previous case-studies demonstrated the effectiveness of prednisone and IVIg therapy on IRAb-induced autoimmune hypoglycaemia (**Supplemental Table 1**), both treatments only transiently alleviated the hypoglycaemic symptoms in our patient. After almost a year of supportive treatment, the hypoglycaemic symptoms were significantly reduced in this patient, consistent with previous report that spontaneous remission might occur in some cases (15). A small prospective cohort study had demonstrated that combined immunosuppressive therapy with rituximab, high-dose pulsed steroids, and cyclophosphamide induced remission in patients with hyperglycaemia and severe insulin resistance induced by IRAb (20). However, whether this treatment protocol is also useful for patients with IRAb-induced hypoglycaemia alone remains to be investigated. Moreover, many patients are intolerant with immunosuppressive therapies and hence more targeted treatment for IRAb are eagerly awaited.

The heterogeneity in the clinical manifestations caused by IRAb may be related to its functional complexity and polyclonal characteristics (3). In our study, we found that the IRAb from our patient exerted insulin-like effects and acted as a robust agonist for insulin receptor signaling causing severe hypoglycaemia. However, in the more typical TBIRS patients who present with severe insulin resistance and hyperglycaemia (20), we speculated that their IRAb possibly functions in a distinct way that it only binds with the insulin receptor but is unable to activate insulin receptor signaling in a similar fashion to insulin. In other words, their IRAb occupies the insulin-binding domain of the insulin receptor, rendering their endogenous insulin to become ineffective in binding with the insulin receptors and hence causing severe insulin resistance. Furthermore, it should be noted that many patients with IRAb had hyperinsulinaemia without excessively high serum C-peptide levels, suggesting that IRAb might also have inhibitory effects on degradation of endogenous insulin. Insulin degradation is initiated by binding of insulin with the insulin receptor, followed by endocytosis of the insulin-insulin receptor complex and direction to the intracellular lysosomes for further degradation (11). In this regard, studies by us and the others have identified that IRAb could significantly inhibit insulin binding with insulin receptor on cell surface and may therefore disrupt normal insulin degradation (21), leading to hyperinsulinaemia. Nonetheless, the exact molecular mechanism on how IRAb disrupts insulin action requires clarification in further studies.

In this study, we have developed an easy-to-use ELISA kit for IRAb measurements according to the sandwich principle (22), using capture antibody and purified antigen to bind with IRAb in samples, followed by IRAb detection using HRP-conjugated anti-human IgG (**Figure 5A**). Indirect ELISA method was initially employed to detect IRAb, in which the purified antigen was directly coated at the bottom of wells to capture IRAb in samples. However, this was limited by the strong and noisy background signals. Therefore, a rabbit anti-hIR capture antibody was added to overcome these background signals likely induced by the direct coating antigen. Importantly, the IRAb titers of our patient measured at different time points using this ELISA matched closely with those measured using IP assays, and the S/N value was satisfactory to discriminate between the positive and negative samples.

A major limitation of this study is that clinical samples were only available from one IRAb-positive patient, even though we were able to correlate his serial IRAb changes with clinical status. Hence it is not possible for us to accurately determine the sensitivity, specificity and the exact COV of this ELISA kit using receiver-operating characteristic curves or report the results with exact antibody titers (23). More collaborative studies with researchers and clinicians from other endocrine centres are needed to validate the performance characteristics of this newly-developed ELISA and establish an international IRAb standardization program.

From a clinical point of view, there remains a possibility that some patients who have type 2 diabetes but with marked hyperglycaemia and severe insulin resistance, or those with unexplained refractory hypoglycaemia, may harbour IRAb which are underdiagnosed due to a lack of commercially available testing for this autoantibody. It would be of potential clinical impact, using our user-friendly ELISA kit, to investigate the prevalence of IRAb in patients with severe dysglycaemia or insulin resistance. Moreover, our ELISA kit can be used to monitor treatment responses and guide therapeutic strategies in those rare TBIRS patients whose fluctuating glycaemia can sometimes be very difficult to manage.

## Data availability statement

The original contributions presented in the study are included in the article/**Supplementary Material**. Further inquiries can be directed to the corresponding authors.

## Ethics statement

The studies involving human participants were reviewed and approved by Institutional Review Board of The University of Hong Kong/Hospital Authority Hong Kong West Cluster. The patients/participants provided their written informed consent to participate in this study.

## Author contributions

All authors contributed to the study conception and design. Material preparation, data collection and analysis were performed by LG, C-LW, BL, YL and HH. The first draft of the manuscript was written by LG and C-LW, and all authors commented on previous versions of the manuscript. All authors contributed to the article and approved the submitted version.

## Funding

This project was supported by the General Research Fund (17121819) and Area of Excellence (AOE/M/707-18) from the Research Grant Council of Hong Kong.

## Conflict of interest

The authors declare that the research was conducted in the absence of any commercial or financial relationships that could be construed as a potential conflict of interest.

## Publisher’s note

All claims expressed in this article are solely those of the authors and do not necessarily represent those of their affiliated organizations, or those of the publisher, the editors and the reviewers. Any product that may be evaluated in this article, or claim that may be made by its manufacturer, is not guaranteed or endorsed by the publisher.
