# Supplementary material for: Development of a novel diagnostic assay for insulin receptor autoantibodies based on a patient with autoimmune hypoglycaemia
Source: Front Endocrinol (Lausanne). 2022 Oct 25;13:1029297. doi: 10.3389/fendo.2022.1029297 (PMC9642853; doi:10.3389/fendo.2022.1029297)
Supplement: Supplementary file 1 [file Table_1.pdf]

**Supplemental Table 1. Previous cases of autoimmune hypoglycaemia caused by autoantibodies against the insulin receptor.**

| Patient | Year* | Country/ Region | Race | Gender | Age | Acanthosis nigricans | Fasting insulin | Fasting C-peptide | Underlying disease/ Autoantibody | Clinical course | Ref |
|---|---|---|---|---|---|---|---|---|---|---|---|
| 1 | 1978 | US | Creole | F | 37 | Yes | High | – | TBIR/ IRAb | Spontaneous clearing of the acanthosis nigricans over 3 years; Hypoglycaemia persisted and controlled by frequent feeding | [1, 2] |
| 2 | 1978 | US | Black | F | 38 | Yes | – | – | TBIR, Nephrotic syndrome/ IRAb, ANA | No remission by immunosuppressive therapy and died from hypoglycaemia after 23 months | [1, 2] |
| 3 | 1982 | US | Black | F | 60 | No | Normal | – | TBIR, Hashimoto's thyroiditis/ ANA, IRAb, TPO Ab | Hypoglycaemia remitted by prednisolone therapy but died from the sudden onset of abdominal pain, nausea and vomiting | [3] |
| 4 | 1983 | Italy | – | F | 46 | No | High | – | TBIR, Lupus nephritis/ IRAb | Plasmapheresis improved the patient's metabolic status | [4] |
| 5 | 1987 | Israel | – | M | 1 | No | Low | Low | AIH/ IRAb | Hypoglycaemia remitted by prednisolone therapy | [5] |
| 6 | 1987 | US | Black | F | 43 | Yes | High | – | AIH, TBIR/ IRAb | Euglycemia obtained by treatment with prednisolone and cyclophosphamide | [6] |
| 7 | 1987 | UK | – | F | 50 | No | Low | Low | Hodgkin's disease, AIHA/ IRAb | No change by frequent carbohydrate meals; Died from splenomegaly and chemotherapy later | [7] |
| 8 | 1987 | UK | – | M | 76 | No | Low | Low | Hodgkin's disease/ IRAb | Poor response to plasmapheresis and azathioprine; Hypoglycaemia remitted by prednisolone after 8-month therapy; Died from deteriorated liver function later | [8] |

| | | | | | | | | | | | |
|---|---|---|---|---|---|---|---|---|---|---|---|
| 9 | 1988 | US | Black | F | 52 | No | Low | Low | SLE/ IRAb, ANA, DNA Ab | Hypoglycaemia remitted by prednisolone therapy | [9] |
| 10 | 1989 | France | — | M | 1 | No | — | — | AIH, AIHA, SCID/ IRAb | Hypoglycaemia remitted by frequent feeding and prednisolone therapy in 15 days | [10] |
| 11 | 1989 | US | — | F | 24 | No | Low | Low | SLE/ IRAb, ANA, nRNP Ab, SM Ab | Hypoglycaemia remitted by prednisolone therapy | [11] |
| 12 | 1989 | US | Black | F | 23 | Yes | High | — | DM, TBIR/ IRAb, IAA, ANA | Died from cardiopulmonary arrest | [12] |
| 13 | 1989 | Japan | Asian | M | 62 | Yes | High | Low | TBIR/ IRAb, ANA, nRNP Ab, SM Ab | Not improved by immunosuppressive therapy with cyclophosphamide, prednisolone and methylprednisolone; Discharged with dietary instructions recommending frequent food intake | [13, 14] |
| 14 | 1990 | Hong Kong | Asian | M | 49 | No | Normal | — | Hodgkin's disease/ IRAb | Hypoglycaemia remitted by prednisone therapy within 4 weeks | [15] |
| 15 | 1991 | Italy | White | F | 59 | No | Low | Low | SLE, TBIR/ IRAb, ANA, dsDNA Ab | Hypoglycaemia remitted after 10 days of prednisolone therapy | [16] |
| 16 | 1994 | US | Black | M | 59 | — | — | — | TBIR, AIH/ IRAb | Died from severe hypoglycaemia with unconsciousness and dysfunction of multiple organs | [17] |
| 17 | 1998 | US | — | F | 44 | Yes | High | High | TBIR, AIH, Suspicious SLE/ IRAb, ANA | Euglycemia obtained after treatment of dextrose | [18] |
| 18 | 2000 | Japan | Asian | F | 50 | Yes | High | High | SLE, TBIR, AIH/ IRAb, ANA, dsDNA Ab | Euglycemia obtained after treatment of prednisolone | [19] |

| | | | | | | | | | | | |
|---|---|---|---|---|---|---|---|---|---|---|---|
| 19 | 2006 | France | White | M | 88 | No | High | Normal | Arterial hypertension/ IRAb, IAA | Hypoglycaemia remitted promptly by prednisolone therapy | [20] |
| 20 | 2007 | US | Black | F | 61 | Yes | — | High | TBIR, AIH/ ANA, TPO Ab, TgAb, IRAb | Euglycemia obtained by treatment with prednisolone and methylprednisolone | [21] |
| 21 | 2007 | Japan | Asian | F | 74 | No | High | High | TBIR, AIH/ IRAb, TgAb, IAA | Hypoglycaemia remitted by prednisolone therapy | [22] |
| 22 | 2008 | Japan | Asian | F | 23 | Yes | High | High | SLE, TBIR, AIH/ IRAb, ANA | Euglycemia obtained by treatment with steroid pulse and subsequent high dose of prednisolone; Later died from hemorrhagic shock due to duodenal ulcer and subsequent pneumocystis carinii pneumonia | [23] |
| 23 | 2008 | Japan | Asian | F | 40 | No | Normal | Normal | SLE, TBIR/ ANA, dsDNA Ab, ssDNA Ab, SSA Ab | Hypoglycaemia remitted after 2.5 month of prednisolone therapy | [24] |
| 24 | 2009 | China | Asian | M | 37 | No | High | High | SLE, AIH/ IRAb, ANA, dsDNA Ab, SM Ab, IAA | SLE and hypoglycaemia remitted by treatment with prednisone, chloroquine, intravenous cyclophosphamide for 6 months | [25] |
| 25 | 2009 | India | Asian | F | 50 | Yes | — | — | SLE, TBIR, AIH/ IRAb, ANA | Hyperglycemia corrected by a pulse therapy with intravenous methylprednisolone for 3 days with maintenance dose of steroids and azathioprine; Mild hypoglycaemia controlled by frequent meals | [26] |

| | | | | | | | | | | | |
|---|---|---|---|---|---|---|---|---|---|---|---|
| 26 | 2011 | Japan | Asian | F | 32 | No | Low | Low | AIH/ IRAb, TgAb, Microsome Ab, Platelet Ab | Immediately remitted after delivery | [27] |
| 27 | 2011 | South Korea | — | M | 35 | Yes | Normal | Low | AIH/ IRAb | No response to plasmapheresis; Hypoglycaemia and acanthosis remitted by prednisolone and azathioprine after 17-month therapy | [28] |
| 28 | 2013 | Japan | Asian | F | 72 | No | — | Low | T2D/ IRAb, IAA | Stable glycemia maintained at approximately 7.0% with liraglutide (0.9 mg/day) and gliclazide (20 mg/day) | [29] |
| 29 | 2013 | France | — | F | 47 | Yes | Low | Low | DM/ IRAb | Hypoglycaemia remitted by prednisolone after 5-month therapy; persistent hyperglycemia controlled by insulin therapy | [30] |
| 30 | 2013 | South Korea | Asian | F | 38 | No | High | High | SLE, ILD/ IRAb, ANA, dsDNA Ab, SSA Ab, SSB Ab, nRNP Ab | Hypoglycaemia remitted by immunosuppressive therapy including prednisolone, hydroxychloroquine and azathioprine | [28] |
| 31 | 2014 | France | White | M | 44 | Yes | High | Normal | SLE, T2D, Acanthosis nigricans/ IRAb, ANA, nRNP Ab | Hypoglycaemia remitted by rituximab after 10-month therapy | [31] |
| 32 | 2017 | UK | Latina | F | 8 | Yes | High | High | T1D, SLE, Dermatomyositis, Autoimmune thyroiditis/ IRAb, ANA, dsDNA Ab, GAD65 Ab, TPO Ab, TgAb | Poor response to B-cell immunomodulation, including high-dose corticosteroids, mycophenolate mofetil, rituximab, IVIg, and plasmapheresis; Hypoglycaemia improved by bortezomib | [32] |

| | | | | | | | | | | | |
|---|---|---|---|---|---|---|---|---|---|---|---|
| 33 | 2018 | US | Black | F | 60 | Yes | High | High | SLE, Hashimoto hypothyroidism/ IRAb, TPO Ab | Euglycemia obtained after IVIg treatment | [33, 34] |
| 34 | 2021 | Japan | Asian | M | 59 | Yes | High | Normal | SLE, Lupus nephritis/ IRAb, ANA, Ro/SSA Ab | Hypoglycaemia remitted by rituximab and glucocorticoids | [35] |
| 35 | 2022 | US | White | F | 55 | No | Low | Low | TBIR, Mixed connective tissue disease/ IRAb, Sm Ab, U1RNP Ab | Hypoglycaemia remitted by glucose therapy and nocturnal nasogastric feeding | [36] |
| 36 | 2022 | New Zealand | – | M | 55 | No | Low | Low | Cardiovascular, Peripheral vascular disease, Hypertension, Dyslipidaemia/ IRAb, ANA, Ro52 Ab, AM2 Ab | No further relapse of SLE disease activity or any hypoglycemic episodes after 18 weeks the multitarget therapy; Died from a severe cerebral infarction on Day 180 | [37] |

*, year of publication; -, unknown or not determined; AIH, autoimmune hypoglycaemia; AIHA, autoimmune haemolytic anaemia; AM2 Ab, anti-adrenomedullin 2 antibody; ANA, anti-nuclear antibody; DM, diabetes mellitus; dsDNA Ab, anti-double strand DNA antibody; GAD65 Ab, anti-glutamic acid decarboxylase autoantibody; IAA, insulin autoantibody; ILD, interstitial long disease; IRAb, autoantibodies against insulin receptor; IVIg, intravenous immunoglobulin; nRNP Ab, anti-nuclear ribonucleoprotein antibody; SCID, severe combined immunodeficiency; SLE, systemic lupus erythematosus; SM Ab, anti-Smith antibody; SSA Ab, anti–Sjogren syndrome antigen A autoantibody; SSB Ab, anti-Sjogren syndrome antigen B autoantibody; T1D, type 1 diabetes; T2D, type 2 diabetes; TBIR, type B insulin resistance; TgAb, thyroglobulin antibody; TPO Ab, thyroid peroxidase antibody; U1RNP Ab, anti-U1 ribonucleoprotein antibodies.

**Reference：**


1. Kahn, C.R., et al., *The syndromes of insulin resistance and acanthosis nigricans: insulin-receptor disorders in man.* New England Journal of Medicine, 1976. **294**(14): p. 739-745.
2. FLIER, J.S., et al., *The evolving clinical course of patients with insulin receptor autoantibodies: spontaneous remission or receptor proliferation with hypoglycemia.* The Journal of Clinical Endocrinology Metabolism, 1978. **47**(5): p. 985-995.
3. Taylor, S.I., et al., *Hypoglycemia associated with antibodies to the insulin receptor.* New England Journal of Medicine, 1982. **307**(23): p. 1422-1426.
4. Tardella, L., et al., *Circulating anti-insulin receptor antibodies in a patient suffering from lupus nephritis and hypoinsulinemic hypoglycaemia.* Journal of clinical laboratory immunology, 1983. **12**(3): p. 159-165.
5. Elias, D., et al., *Antibodies to insulin receptor followed by anti-idiotype: antibodies to insulin in child with hypoglycemia.* Diabetes, 1987. **36**(3): p. 348-354.
6. Sims, R.E., et al., *Successful immunosuppressive therapy in a patient with autoantibodies to insulin receptors and immune complex glomerulonephritis.* Southern Medical Journal, 1987. **80**(7): p. 903-906.
7. Walters, E., et al., *Hypoglycaemia due to an insulin-receptor antibody in Hodgkin's disease.* The Lancet, 1987. **329**(8527): p. 241-243.
8. Braund, W., et al., *Autoimmunity to insulin receptor and hypoglycaemia in patient with Hodgkin's disease.* The Lancet, 1987. **329**(8527): p. 237-240.
9. Moller, D.E., et al., *Autoantibodies to the insulin receptor as a cause of autoimmune hypoglycemia in systemic lupus erythematosus.* The American journal of medicine, 1988. **84**(2): p. 334-338.
10. Rochet, N., et al., *Hypoglycaemia induced by antibodies to insulin receptor following a bone marrow transplantation in an immunodeficient child.* Diabetologia, 1989. **32**(3): p. 167-172.
11. Howard, R.L., L.K. Beck, and A. Schneebaum, *Systemic lupus erythematosus presenting as hypoglycemia with insulin receptor antibodies.* Western Journal of Medicine, 1989. **151**(3): p. 324.
12. BLOISE, W., et al., *Atypical antiinsulin receptor antibodies in a patient with type B insulin resistance and scleroderma.* The Journal of Clinical Endocrinology Metabolism, 1989. **68**(1): p. 227-231.
13. Kiyokawa, H., et al., *Hyperinsulinemia due to impaired insulin clearance associated with fasting hypoglycemia and postprandial hyperglycemia: an analysis of a patient with antiinsulin receptor antibodies.* The Journal of Clinical Endocrinology Metabolism, 1989. **69**(3): p. 616-621.
14. Ishimura, E., et al., *Combination of glomerulonephritis with diabetic glomerulopathy in a patient with diabetes mellitus due to autoantibody to insulin receptor.* Nephron, 1994. **66**(1): p. 105-107.
15. Chan, J.C., et al., *Hypoglycaemia and Hodgkin's disease.* British journal of haematology, 1990. **76**(3): p. 434-436.
16. PAOLO, S.D. and R. GIORGINO, *Insulin resistance and hypoglycemia in a patient with systemic lupus erythematosus: description of antiinsulin receptor antibodies*

*that enhance insulin binding and inhibit insulin action.* The Journal of Clinical Endocrinology Metabolism, 1991. **73**(3): p. 650-657.

17. O'Brien, T.D., et al., *Islet amyloidosis in a patient with chronic massive insulin resistance due to antiinsulin receptor antibodies.* The Journal of Clinical Endocrinology, 1994. **79**(1): p. 290-292.
18. Freeland, B.S., R.E. Paglia, and D.L. Seal, *Clinical challenges of type B insulin resistance: a case study.* The Diabetes Educator, 1998. **24**(6): p. 728-733.
19. Yamasaki, H., et al., *Anti-insulin receptor autoantibodies in a patient with type B insulin resistance and fasting hypoglycemia.* Acta diabetologica, 2000. **37**(4): p. 189-196.
20. Bortolotti, D., et al., *Spontaneous hypoglycaemia in the presence of both anti-insulin antibody and anti-insulin receptor antibody.* Diabetes metabolism, 2006. **32**(6): p. 598-603.
21. Fareau, G.G., et al., *Regression of acanthosis nigricans correlates with disappearance of anti–insulin receptor autoantibodies and achievement of euglycemia in type B insulin resistance syndrome.* Metabolism, 2007. **56**(5): p. 670-675.
22. Kato, T., et al., *Severe hypoglycaemia in a person with insulin autoimmune syndrome accompanied by insulin receptor anomaly type B.* Diabetic medicine, 2007. **24**(11): p. 1279-1281.
23. Nagayama, Y., et al., *Type B insulin resistance syndrome induced by increased activity of systemic lupus erythematosus in a hemodialysis patient.* Clinical nephrology, 2008. **69**(2): p. 130-134.
24. Kato, Y., Y. Ichiki, and Y. Kitajima, *A case of systemic lupus erythematosus presenting as hypoglycemia due to anti-insulin receptor antibodies.* Rheumatology international, 2008. **29**(1): p. 103-105.
25. Qing, Y., J. Zhou, and G. Yuan, *Systemic lupus erythematosus presenting as hypoglycaemia with insulin receptor antibodies and insulin autoantibodies.* Lupus, 2009. **18**(5): p. 457-459.
26. Ostwal, V. and J. Oak, *Type B insulin resistance in a systemic lupus erythematosus patient.* International Journal of Rheumatic Diseases, 2009. **12**(2): p. 174-176.
27. Toshihiro, M., et al., *Recurrent hypoglycemia during pregnancies in a woman with multiple autoantibodies including anti-insulin receptor antibody and anti-platelet antibody, whose serum lowered murine blood glucose levels and phosphorylated insulin receptor of CHO-IR cells.* Endocrine journal, 2011: p. 1109090623-1109090623.
28. Chon, S., et al., *Autoimmune hypoglycemia in a patient with characterization of insulin receptor autoantibodies.* Diabetes Metabolism 2011. **35**(1): p. 80-85.
29. Kawanami, D., et al., *Successful control of a case of severe insulin allergy with liraglutide.* Journal of Diabetes Investigation, 2013. **4**(1): p. 94-96.
30. Maiza, J.-C., et al., *Anti-insulin receptor antibodies related to hypoglycemia in a previously diabetic patient.* Diabetes Care, 2013. **36**(6): p. e77-e77.
31. Bourron, O., et al., *Type B Insulin-resistance syndrome: a cause of reversible autoimmune hypoglycaemia.* The Lancet, 2014. **384**(9953): p. 1548.
32. Shanker, K., et al., *Intractable hypoglycemia in the setting of autoimmune overlap syndrome.* Pediatrics, 2017. **139**(6).
33. Viswanathan, L. and I. Sirisena, *Immunosuppressive therapy in treatment of refractory hypoglycemia in type B insulin resistance: a case report.* Journal of the Endocrine

Society, 2017. **1**(12): p. 1435-1439.

34. Deng, X., *Type B Insulin Resistance Presenting as Hypoglycemia.* Journal of Endocrinology and Thyroid Research, 2018. **3**.

35. Temmoku, J., et al., *Effect of a multitarget therapy with prednisolone, mycophenolate mofetil, and tacrolimus in a patient with type B insulin resistance syndrome complicated by lupus nephritis.* Mod Rheumatol Case Rep, 2022. **6**(1): p. 41-46.

36. Petersen, M.C., et al., *Insulin Receptor Autoantibody–mediated Hypoglycemia in a Woman With Mixed Connective Tissue Disease.* Journal of the Endocrine Society, 2021.

37. Brown, N., et al., *A typical presentation of type B insulin resistance syndrome with isolated hypoglycaemia and suppressed insulin.* BMJ Case Reports CP, 2022. **15**(2): p. e246523.
